# Production of bioethanol as useful biofuel through the bioconversion of water hyacinth (*Eichhornia crassipes*)

**DOI:** 10.1007/s13205-016-0385-y

**Published:** 2016-02-15

**Authors:** Arpan Das, Priyanka Ghosh, Tanmay Paul, Uma Ghosh, Bikas Ranjan Pati, Keshab Chandra Mondal

**Affiliations:** 1 Department of Microbiology, Maulana Azad College, Kolkata, West Bengal 700013 India; 2 Food Technology and Biochemical Engineering Department, Jadavpur University, Kolkata, West Bengal 700032 India; 3 Department of Microbiology, Vidyasagar University, Midnapore, West Bengal 721102 India

**Keywords:** *Eichhornia crassipes*, Pretreatment, Mixed fermentation, Central composite design, Bioethanol

## Abstract

Water hyacinth (*Eichhornia crassipes*) represents a promising candidate for fuel ethanol production in tropical countries because of their high availability and high biomass yield. Bioconversion of such biomass to bioethanol could be wisely managed through proper technological approach. In this work, pretreatment of water hyacinth (10 %, w/v) with dilute sulfuric acid (2 %, v/v) at high temperature and pressure was integrated in the simulation and economic assessment of the process for further enzymatic saccharification was studied. The maximum sugar yield (425.6 mg/g) through enzymatic saccharification was greatly influenced by the solid content (5 %), cellulase load (30 FPU), incubation time (24 h), temperature (50 °C), and pH (5.5) of the saccharifying medium. Central composite design optimized an ethanol production of 13.6 mg/ml though a mixed fermentation by *Saccharomyces cerevisiae* (MTCC 173) and *Zymomonas mobilis* (MTCC 2428). Thus the experiment imparts an economic value to water hyacinths that are cleared from choking waterways.

## Introduction

The combustion of fossil fuels has created a global anxiety for the environment and world economy. Overuse of fossil fuel is increasing the carbon dioxide level in the atmosphere and significantly contributes to the global warming (Silva et al. 2011; Abdel-Fattah and Abdel-Naby 2012). Countries across the world have directed state policies toward the utilization of biomass for meeting their future energy demands to meet carbon dioxide reduction targets as specified in the Kyoto Protocol as well as to decrease dependence on the supply of fossil fuels (Sarkar et al. 2012). Thus, there is a pressing need to adapt to the use of bioethanol as a renewable and clean energy source. Recently, research has focused on using non-edible biomass as raw materials including lignocelluloses, celluloses, and marine algae rather than the first-generation biomass such as starch and sugar biomass (Demirbas 2010; Ganguly et al. 2012).

Agro residues when used for ethanol production may address this problem to an extent, but the operation of large-scale plants for cellulosic ethanol production still have several limitations, including high capital investment, technical knowledge, and the high transportation costs of feedstock.

In India, water hyacinth (*Eichhornia crassipes*), an aquatic weed, was first observed in West Bengal at the beginning of 1890 and is now present throughout the country except in the more arid western part of Rajasthan, in the rugged regions of the north, and in Kashmir. This tropical plant infests large areas of water resources and consequently leads to reduction of biodiversity, blockage of rivers, and drainage system, depletion of dissolved oxygen, alteration of water chemistry, and involvement in environmental pollution (Guragain et al. 2011). The plant tolerates extremes in water level fluctuations, seasonal variations in flow velocity, nutrient availability, pH, temperature, and toxic substances (Ganguly et al. 2012). The utilization of water hyacinth as the feedstock for bioethanol production has a number of advantages. Water hyacinth is low in lignin content with high amounts of cellulose and hemicellulose (Poddar et al. 1991; Gressel 2008). This lignocellulose can more easily be bio-converted by enzymatic means to fermentable sugar, thus resulting in an enormous amount of utilizable biomass for bioethanol production. In addition, being an aquatic plant, it does not compete with food crops for arable lands. Its very high growth rate, 60–100 ton/ha/year, is also favorable for its commercial cultivation (Mishima et al. 2008). However, as cellulose components are generally covered with lignin and hemicellulose in the cellulosic biomass, it is necessary to degrade and remove lignin as well as hemicellulose from the cellulosic biomass. Thus, the suitable pretreatment method is required to accelerate the saccharification of cellulosic biomass and their bioconversion to ethanol.

Ideally, these pretreatments attempt to (1) minimize the loss of sugars, (2) consume minimum amounts of energy, (3) improve the enzymatic digestibility, (4) reduce the quantity of by-products and fermentation inhibitors, and (5) reduce costs. In this regard production of lignocellulosic bioethanol from the widely available waste biomass like water hyacinth can serve a dual purpose. Besides reduction of fossil fuel scarcity, it can control environmental pollution and accelerate rural development. As in most developing countries, the majority of India’s labor force works in the agricultural sector; therefore, in India there is particularly high potential for bioethanol to raise incomes, provide employment, and contribute to rural development.

## Materials and methods

### Microorganism and cultural condition

Fungal strain *A. fumigatus* ABK9 (GenBank Acc. No-HM807348.1) was pre isolated from the soil and used in the study (Das et al. 2013a). The strain was grown on potato dextrose agar (PDA) slants at 30 °C for 5 days (until good sporulation occurred) and stored at 4 °C until use. *Saccharomyces cerevisiae* (MTCC 173) and *Zymomonas mobilis* (MTCC 2428), two distillery strains for ethanol production were purchased from the Microbial Type Culture Collection (MTCC), Chandigarh, India. The *S. cerevisiae* culture was grown in YEP broth media [contained (w/v) yeast extract 0.3 %, peptone 1.0 %, dextrose 2 %, pH 6.0] and *Z. mobilis* was grown in nutrient-rich medium containing dextrose 2 %, yeast extract 1.0 %, KH_2_PO_4_ 0.2 %, and pH 6.0. After incubation for 24 h at 120 rpm, they were used as inoculum for alcohol production.

### Raw material

Fresh water hyacinth (*Eichhornia crassipes*) was collected from local ponds and washed to remove adhering dirt, chopped into small pieces (about 1 cm in length), and air-dried. The samples were ground, and the particles of size between 0.45 and 0.9 mm were prepared for the following pretreatments.

### Effect of sample pretreatment and biomass loading

Various pre-hydrolysis treatments were investigated, including dilute acid (H_2_SO_4_, HNO_3_, HCl), alkali (NaOH), and heat treatment or combinations of two of them applied consecutively (Table 1). 10 g of biomass and either dilute acid (2 %) or NaOH (2 %) were mixed at a solid/liquid ratio of 1:10 and kept at room temperature (40 °C) for 60 min. The acid- or alkali-soaked samples were drained, washed with distilled water to neutralize the pH, and then air-dried. Additionally, different pre-treatment methods were combined by treating the biomass individually with acid and alkali under steam treatment at a constant temperature (121 °C) for 60 min. Following steam treatment, the samples were washed with water as before. Biomass loading during pretreatment was also optimized by adjusting various solid liquid ratios (2–15 %, w/w).

**Table 1:** The effect of different combinational pretreatments of dried water hyacinth biomass

Exp. no	Acid treatment						Alkali treatment		Heat treatment		Reducing sugar loss (mg/g)	Glucose loss (mg/g)	Cellulose (%)	Hemicellulose (%)	Lignin (%)
	HNO_3_ (%)	Time (min)	HCl (%)	Time (min)	H_2_SO_4_ (%)	Time (min)	NaOH (%)	Time (min)	Temperature (°C)	Time (min)					
1	2	60	–	–	–	–	–	–	–	–	104	51.2	27.2	28.2	2.3
2	2	–	–	–	–	–	–	–	121	60	129	77.5	32.6	24.3	1.3
3	–	–	2	60	–	–	–	–	–	–	98	47.8	26.5	29.6	2.8
4	–	–	2	–	–	–	–	–	121	60	124	74.6	31.9	26.6	1.2
5	–	–	–	–	2	60	–	–	–	–	110	56.3	28.3	24.1	2.7
6	–	–	–	–	2	–	–	–	121	60	149	88.8	35.4	19.6	0.9
7	–	–	–	–	–	–	2	60	–	–	84	34.6	25.4	33.9	1.8
8	–	–	–	–	–	–	2	–	121	60	112	85.9	28.9	35.3	0.6
Control	–	–	–	–	–	–	–	–	–	–	0.2	0.04	24.7	32.2	3.2

### Scanning electron microscopy

The structural changes in the morphology of WH before and after pretreatment were studied by scanning electron microscope (JEOL JSM-5600). Images were taken at a magnification of 1000×. The specimens were mounted on a conductive tape and coated with gold palladium using a JEOL–JFC-1200 fine coater and observed using a voltage of 25 kV.

### Optimization of enzymatic saccharification

Enzymatic saccharification of pretreated water hyacinth was performed at varying cellulase concentrations (10–30 FPU/g), pH (4.0–8.0), temperatures (40–55 °C) and substrate concentrations (5–30 %, w/v) on a rotary shaker at 100 rpm for 48 h. Samples were withdrawn periodically and the amount of reducing sugar and glucose released was estimated. The hydrolysate was concentrated up to 5 % glucose concentration and subsequently fermented for ethanol production.

### Central composite design for ethanol production

In the study, central composite design (CCD) (Jabasingh and Nachiyar 2011) was used to evaluate the main and interaction effects of the four fermentation factors for ethanol production, namely at A: fermentation time (h), B: fermentation pH, and C: Saccharomyces to Zymomonas ratio. The CCD used in this experiment had six replicates at the central point as well as two replicates at the axial and factorial points (=1.68) leading to 20 experiments. Both linear and quadratic effects as well as the possible interactions of the three variables were calculated and their significances were evaluated by variance analysis (ANOVA). 3D surface plots were drawn to show the effects of independent variables on the response. The ‘fit of the model’ was evaluated by determination of R^2^ coefficient. Regression analysis and estimation of the coefficients were performed using Design Expert software (Stat ease Corp, USA).

### Analytical methods

The cellulase enzyme activity of the culture supernatant was determined by the method described by Wood and Bhat (1988) and expressed in filter paper unit (FPU). One unit (FPU) of enzyme activity was defined as the amount of enzyme that releases 1 µmol of reducing sugar in 1 min under standard condition.

The lignin, hemicellulose, and cellulose content of the WH were analyzed according to the method of Pierre et al. (2011).

The free glucose was determined by GOD-POD method using commercially available glucose oxidase–peroxidase–chromogen reagent (Bergmeyer 1974).

The concentration of total reducing sugars and ethanol was determined using the DNS method (Miller 1959) and dichromate method (AOAC 1990), respectively.

## Result and discussion

### Chemical components of water hyacinth

The average lignocellulosic composition of water hyacinth is as follows(as total percentage of solids): cellulose: 24.7 ± 0.4, hemicellulose: 32.2 ± 0.3, lignin: 3.2 ± 0.2. The cellulose content is in accordance with the data reported by other investigators, while the hemicellulose content is little varied (Klass and Ghosh 1981; Nigam 2002; Kumar et al. 2009). These differences might originate from different geographical locations and different growth state of water hyacinth.

### Pretreatment of water hyacinth

**Fig. 1:**
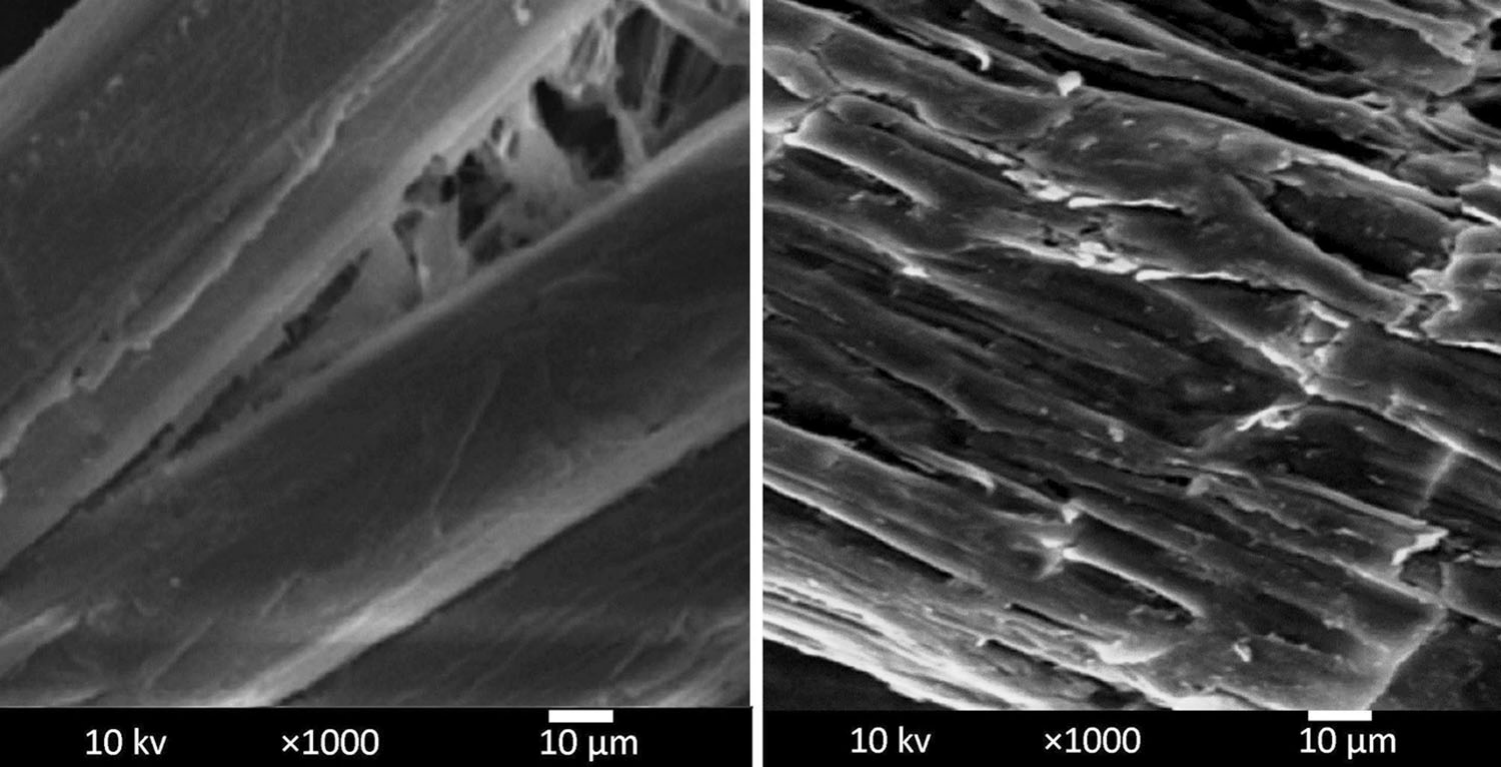
Scanning electron microscopy of untreated and acid (H_2_SO_4_) pretreated water hyacinth

Pretreatment typically breaks down the macroscopic rigidity of the biomass and reduces the physical barriers to mass transport (Liu et al. 2009). In the present study, combinational effects of acid, alkali and temperature with pressure (15 ψ) were compared for their effect on water hyacinth pretreatment. Among the different procedures, acid hydrolysis by H_2_SO_4_ at high temperature seemed to accomplish a considerably higher improvement in pretreatment over heat and alkaline treatments. Lower hemicellulose (19.6 %) and higher residual cellulose (35.4 %) contents indicate that acid treatment had removed most of the hemicellulose and exposed the cellulose for further enzymatic hydrolysis for bioethanol production. Scanning electron microscopy also revealed the loss of structural integrity of the water hyacinth after H_2_SO_4_ treatment (Fig. 1). The acid pretreatments are effective methods used for water hyacinth for dissolving hemicellulose and retaining most of the cellulose (Kumar et al. 2009). Though the conventional acid pretreatments are energy-intensive and environment-unfriendly, the dilute acid at high temperature and pressure is usually applied (Mishima et al. 2008; Masami et al. 2008). The conversion of hemicellulose during dilute acid pretreatment is predictable and has been reported before by several authors when examining the hydrolysis of agricultural residues like corn stover and wheat straw (Lu and Mosier 2007; Kootstra et al. 2009; Kabel et al. 2007). Similar observations were earlier reported for H_2_SO_4_ pretreatment of water hyacinth by Satyanagalakshmi et al. (2011) and sugarcane bagasse pretreatment by Martin et al. (2007). Dilute acid prehydrolysis resulted in 2.7- to 3.7-fold increase for the enzymatic convertibility of water hyacinth and sugarcane bagasse.

### Effect of biomass loading on chemical pretreatment

Studies on the effect of biomass loading on acid pretreatment of water hyacinth showed that biomass loading from 8 to 10 % w/v gave almost same reducing sugar yield, although the residual cellulose (%) increased marginally with an increase in biomass loading and maximum was at 10 % w/w (35.4 %); beyond that loading, there was reduction in reducing sugar yield and % of residual cellulose. The decrease in efficiency of pretreatment above 10 % w/w biomass loading could be due to decrease in the accessibility of the pretreatment agent (H_2_SO_4_). High solid loading has several advantages: it decreases the process cost by lowering the reactor size and energy requirements during the pretreatment and produces a more concentrated product stream (Kootstra et al. 2009). In dilute acid pretreatment, solid loading could usually vary from 5 to 15 % dry lignocellulosic biomass as reported by Kim et al. (2008).

### Optimization of saccharification temperature and pH

**Fig. 2:**
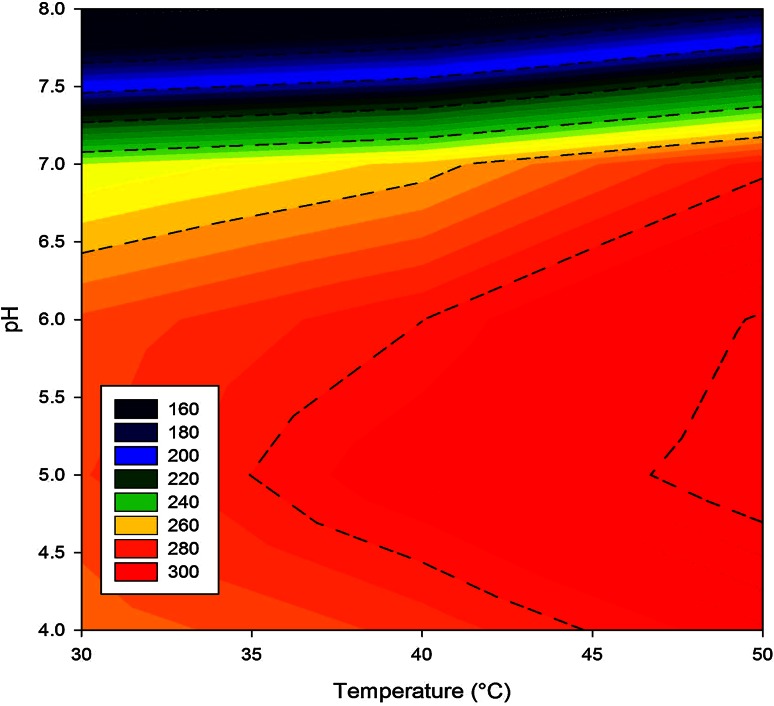
The effect of pH and temperature on enzymatic saccharification of water hyacinth biomass

Initial experiments were done to select the best condition of each pretreatment method and also to compare the effectiveness of different pretreatment methods. Result of the effect of temperature and pH on enzymatic saccharification is shown in Fig. 2. The contour plot indicated that the cumulative outcome of high temperature (50 °C) and acidic pH (5.0–5.5) had a profound effect on pretreated biomass saccharification.

### Effect of incubation time and substrate load on biomass saccharification

**Fig. 3:**
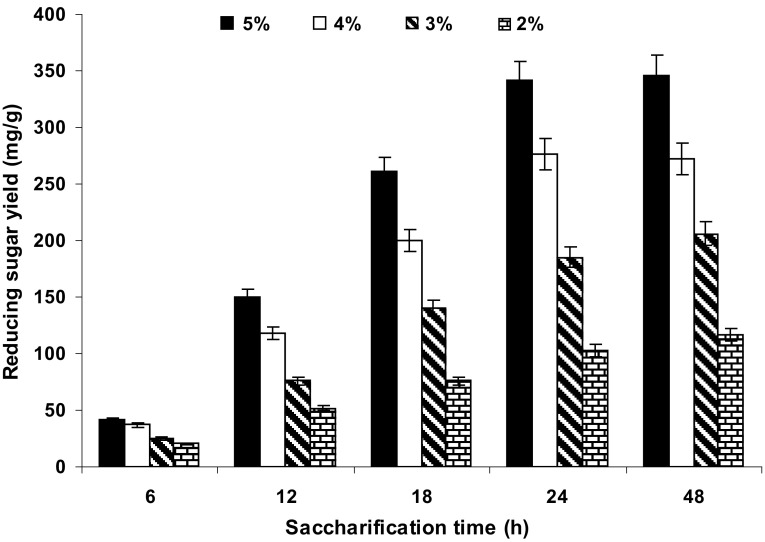
The effect of retention time on enzymatic saccharification of water hyacinth biomass

**Fig. 4:**
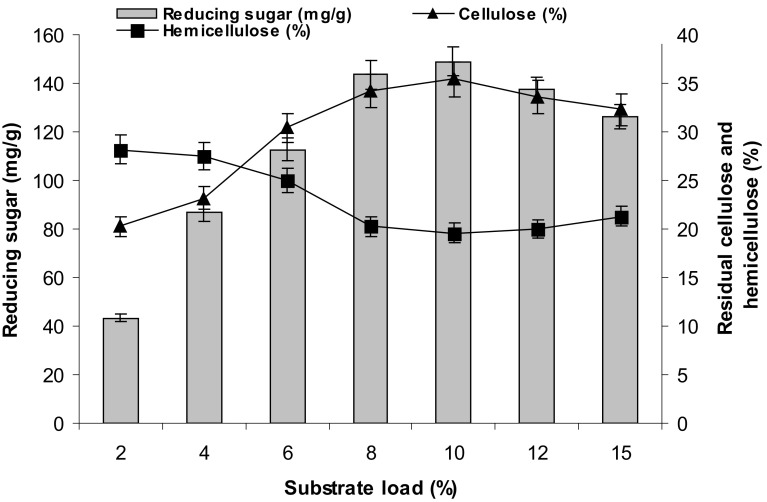
The Effect of substrate (dried water hyacinth) load on chemical (H_2_SO_4_) pretreatment

Figure 3 shows that the enzymatic hydrolysis yield of reducing sugars from pretreated water hyacinth sample increased linearly with incubation time until 24 h while its rate of increment reduced considerably thereafter. A similar result was reported during enzymatic saccharification of water hyacinth cellulose (Abdel-Fattah and Abdel-Naby 2012). On this basis, the incubation time of 24 h was considered the best time period for the enzymatic saccharification. It was further found that the yield of reducing sugar increased gradually along with the substrate load (5 %), indicating that the initial substrate load was also a significant factor for enzymatic saccharification (Fig. 4). Increased solid loading reduces amount of liquid phase per amount of feedstock, leading to low energy demand and low reaction volume, which would reduce the cost of bioethanol production and further downstream processing.

### Effect of enzyme load on biomass saccharification

**Fig. 5:**
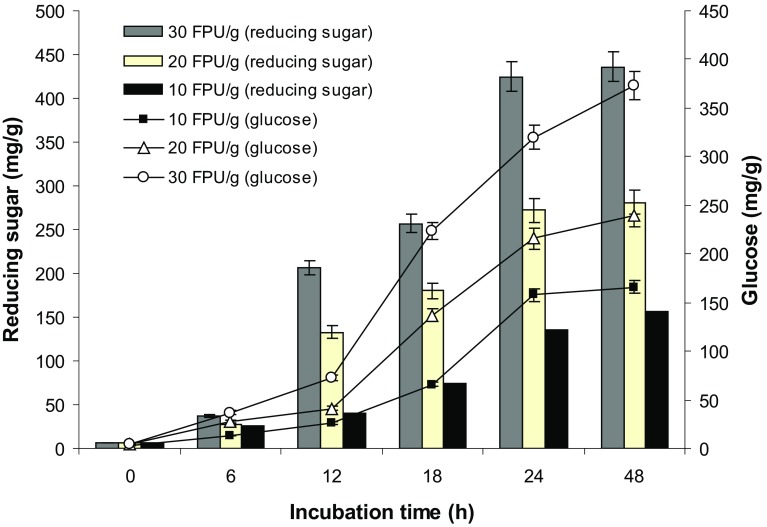
The effect of enzyme load on bio saccharification of water hyacinth biomass

Celluloses have specific domains for binding to their substrate so that the enzyme sits on the polymer and causes a slow degradation (Lynd et al. 2002). For this purpose, initial experiments were conducted to select the optimum enzyme concentration during bio-saccharification. Trials done on WH saccharification indicated that the yield of reducing sugar and glucose was better with higher enzyme loading (30 FPU/g) and it was speculated that an increase in enzyme loading might improve the saccharification efficiency. From Fig. 5, it was found that the hydrolysis yield of reducing sugar and glucose increased with increase in enzyme concentration from 10 to 30 FPU/g of pretreated water hyacinth. After 24 h of saccharification, no superior improvement of reducing sugar and glucose production was observed. Therefore, during enzymatic treatment, 24 h saccharification with an enzyme load of 30 FPU/g of pretreated biomass was selected as the standard condition.

### Central composite design (CCD)

**Fig. 6:**
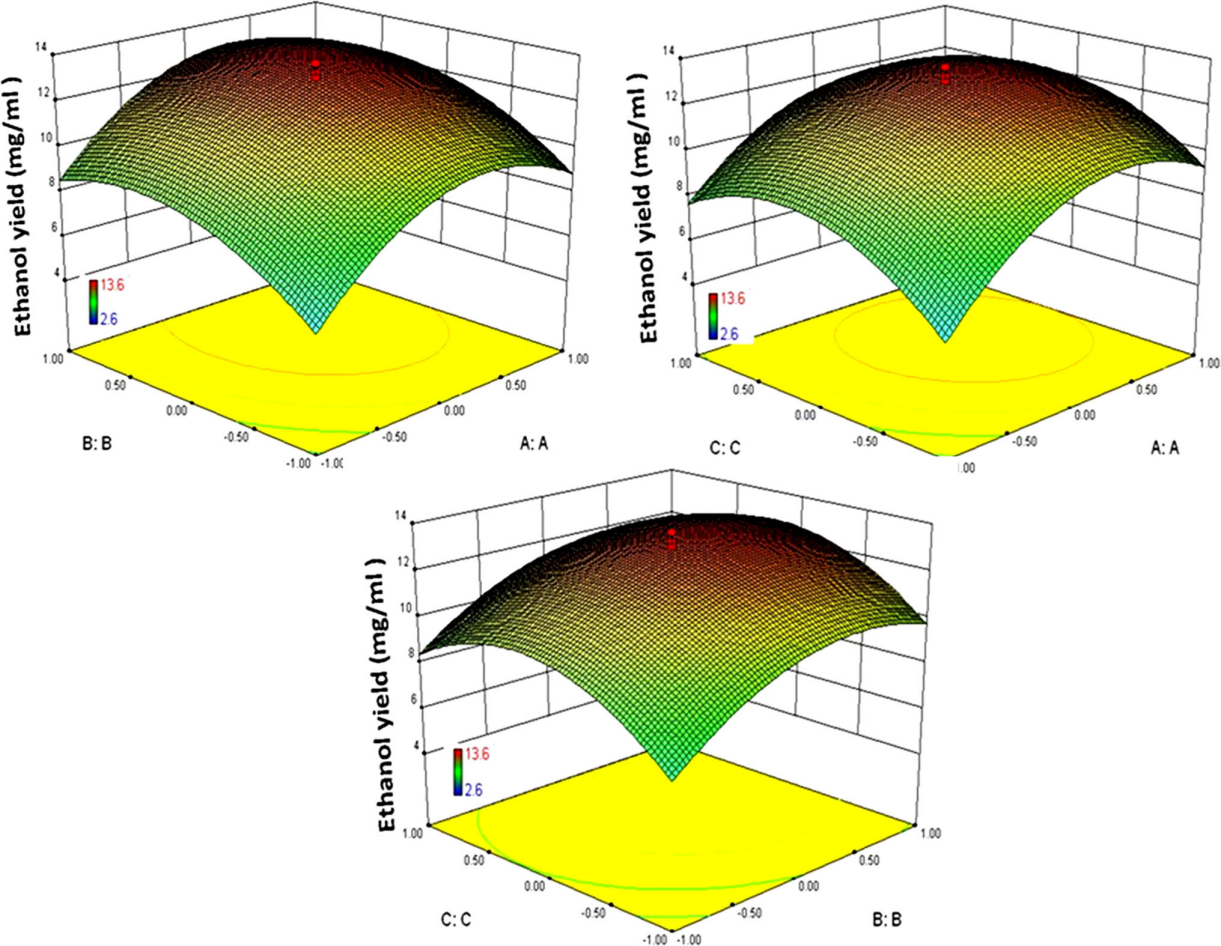
Response surface plot for different combitional effects of the factors on ethanol production by mixed fermentation of *S. cerevisiae* and *Z. mobilis* through central composite design

The optimum levels of the selected factors and the effects of their interactions in ethanol production were determined by the CCD and shown in Table 2. The data were analyzed by multiple regression analysis and a second-order polynomial equation was derived to represent the ethanol production as a function of the independent variables tested:

$$ Y_{\text{ethanol}} = 12.99 + 1.55{\text{A}} + 1.41{\text{B}} + 0.74{\text{C}} + \, 0.0002{\text{AB}} - 0.32{\text{AC}} - 0.075{\text{BC}} - 2.57{\text{A}}^{2} - 1.72{\text{B}}^{2} - 2.25{\text{C}}^{2} , $$

where *Y* represents ethanol production (U/g); A, B, C are fermentation time (h), fermentation pH, and Saccharomyces to Zymomonas ratio respectively. The obtained results were analyzed by analysis of variance (ANOVA) and the predicted as well as observed responses are presented in Table 3. ANOVA of the quadratic regression model suggests that the model is significant with a computed F value of 49.12 and a *P* > *F* lower than 0.05. The value of multiple correlation co-efficient (*R* ^2^) was 0.9779, indicating a better correlation between the observed and predicted values. A lower value for the coefficient of variation suggests higher reliability of the experiment and in this case the obtained CV value of 8.76 % demonstrated a greater reliability of the trials. The ‘adequate precision’ value of 37.82 indicated an adequate signal and suggested that the model can be used to navigate the design space. Response surface curves (Fig. 6) also indicated the interaction effects of variables and for identifying the optimal levels of each parameter for attaining maximal ethanol yield.

**Table 2:** Central composite design along with observed and predicted results for ethanol production

Run	A. Fermentation time (h)	B. Fermentation pH	C. Saccharomyces:Zymomonas	Ethanol yield (mg/ml)	
				Observed response	Predicted response
1	0 (36)	1.68 (8.68)	0 (1)	11.6	10.5
2	1 (42)	−1 (5)	−1 (0.5)	5.8	6.1
3	0 (36)	0 (6)	0 (1)	13.6	13.0
4	0 (36)	0 (6)	1.68 (0.8)	8.5	7.9
5	0 (36)	0 (6)	0 (1)	12.9	13.0
6	0 (36)	−1.68 (3.32)	0 (1)	5.6	5.7
7	1 (42)	−1 (5)	1 (1.5)	7.3	7.1
8	1 (42)	1 (7)	−1 (0.5)	8.9	9.1
9	−1.68 (26.4)	0 (6)	0 (1)	3.7	3.1
10	−1 (30)	−1 (5)	−1 (0.5)	2.6	2.3
11	−1 (30)	1 (7)	1 (1.5)	6.9	7.3
12	0 (36)	0 (6)	0 (1)	12.8	13.0
13	−1 (30)	1 (7)	−1 (0.5)	4.4	5.3
14	0 (36)	0 (6)	0 (1)	13.2	13.0
15	0 (36)	0 (6)	−1.68 (0.7)	5.7	5.8
16	−1 (30)	−1 (5)	1 (1.5)	4.1	4.6
17	0 (36)	0 (6)	0 (1)	12.3	13.0
18	1.68 (45.6)	0 (6)	0 (1)	8.7	8.3
19	0 (36)	0 (6)	0 (1)	13.0	13.0
20	1 (42)	1 (7)	1 (1.5)	8.8	9.8

**Table 3:** ANOVA results of the central composite design for ethanol production

Source	SS^a^	DF^b^	*F* value	Prob > *F*
Model	246.52	9	49.12	<0.0001
A	32.94	1	59.07	<0.0001
B	27.25	1	48.87	<0.0001
C	7.48	1	13.42	0.0044
AB	0.000	1	0.000	1.000
AC	0.84	1	1.52	0.2456
BC	0.045	1	0.081	0.7821
A^2^	95.41	1	171.12	<0.0001
B^2^	42.86	1	76.87	<0.0001
C^2^	73.27	1	131.41	<0.0001
Residual	5.58	10		
Lack of fit	4.64	5	4.97	0.0515
Pure error	0.93	5		
Cor total	252.09	19		

PRESS = 37.82, *R* ^2^ = 0.9779 Adj *R* ^2^ = 0.9580

Pred *R* ^2^ = 0.8500

### Optimization and confirmation experiments

Using Design Expert 8.0.3, numerical optimization subroutine design space was explored with a fitted quadratic model to arrive at the optimum factor concentration. The goals for the variables were set as “in range”, varying from −1 level to +1 level, while for the yield of ethanol production it was set as “maximize.” The optimized variables were found using a desirability objective function that assigns relative importance to the responses. Solutions with higher desirability gave optimum fermentation time of 37.7 h, fermentation pH of 6.41, and *Saccharomyces* to *Zymomonas* ratio of 1. The fermentation with mixed microbial culture were also previously reported for bioethanol production from agricultural wastes (Das et al. 2013b) Under these conditions, confirmation experiments were conducted in three replicates. The observed mean of ethanol production was found to be 13.6 mg/ml, which was largely consistent with the predicted values.

## Conclusion

Water hyacinths were subjected to different pretreatments and among them H_2_SO_4_ pretreatment gave best results. The reducing sugar and glucose yields from enzymatic hydrolysis were maximum at high temperature (50 °C) and acidic pH (5.0–5.5) with 5 % substrate and 30 FPU/g enzyme loading. The concentrated hydrolysate (with 5 % glucose) was subjected for ethanol production through response surface methodology. During co-culture, ethanol production was maximum (13.6 mg/ml) at optimum fermentation time of 37.7 h, fermentation pH of 6.41, and *Saccharomyces* to *Zymomonas* ratio of 1. Water hyacinth is one of the worst weeds in the aquatic ecosystem but it is also a potential resource of biomass available in many tropical regions of the world and with a proper technical knowledge can be used as feedstock for small-scale distributed production of fuel ethanol.
